# The Relative Importance of Spatial and Local Environmental Factors in Determining Beetle Assemblages in the Inner Mongolia Grassland

**DOI:** 10.1371/journal.pone.0154659

**Published:** 2016-05-03

**Authors:** Xiao-Dong Yu, Liang Lü, Feng-Yan Wang, Tian-Hong Luo, Si-Si Zou, Cheng-Bin Wang, Ting-Ting Song, Hong-Zhang Zhou

**Affiliations:** Key Laboratory of Zoological Systematics and Evolution, Institute of Zoology, Chinese Academy of Sciences, Beijing 100101, P. R. China; Universidade de São paulo, BRAZIL

## Abstract

The aim of this paper is to increase understanding of the relative importance of the input of geographic and local environmental factors on richness and composition of epigaeic steppe beetles (Coleoptera: Carabidae and Tenebrionidae) along a geographic (longitudinal/precipitation) gradient in the Inner Mongolia grassland. Specifically, we evaluate the associations of environmental variables representing climate and environmental heterogeneity with beetle assemblages. Beetles were sampled using pitfall traps at 25 sites scattered across the full geographic extent of the study biome in 2011–2012. We used variance partitioning techniques and multi-model selection based on the Akaike information criterion to assess the relative importance of the spatial and environmental variables on beetle assemblages. Species richness and abundance showed unimodal patterns along the geographic gradient. Together with space, climate variables associated with precipitation, water-energy balance and harshness of climate had strong explanatory power in richness pattern. Abundance pattern showed strongest association with variation in temperature and environmental heterogeneity. Climatic factors associated with temperature and precipitation variables and the interaction between climate with space were able to explain a substantial amount of variation in community structure. In addition, the turnover of species increased significantly as geographic distances increased. We confirmed that spatial and local environmental factors worked together to shape epigaeic beetle communities along the geographic gradient in the Inner Mongolia grassland. Moreover, the climate features, especially precipitation, water-energy balance and temperature, and the interaction between climate with space and environmental heterogeneity appeared to play important roles on controlling richness and abundance, and species compositions of epigaeic beetles.

## Introduction

One of the primary goals of ecological surveys are to describe the diversity patterns of species along environmental or geographical gradients and unravel the assembly mechanisms that allow species to coexist across a landscape [1,2]. Often, these investigations lead to important understandings of how communities respond to global change [3,4]. As communities and landscapes are beginning to experience an increasing variety of change, there has been always an interest in the relationship between latitudinal/elevational gradients and species diversity [5,6] and how community structure changes with spatial, temporal or environmental distance in the two decades [7,8]. Insects represent the global majority of terrestrial organisms, and therefore provide a powerful opportunity to study how species distributions are shaped along the geographic gradients [9,10,11,12,13]. Nonetheless, there is considerable idiosyncratic variation across insect taxa, thus it is difficult to draw general conclusions on such a diverse taxonomic group [14]. To further elucidate how insect community structures change along spatial, temporal, and environmental distance, we assessed the shape of diversity patterns in epigaeic beetles inhabiting in temperate arid steppe ecosystems in North China, where detailed ecological analyses of large insect species assemblages are scant [15,16].

Various abiotic and biotic factors have been extensively studied to explain richness and abundance patterns, and can be summarized into six diversity hypotheses such as climate/productivity, environmental heterogeneity, edaphics/nutrients, area, biotic interactions and dispersal/history [17,18,19]. Climate and productivity are usually considered as the most important determinants on species richness at large scales, whereas at finer spatial resolutions, it is difficult isolate a common variable that accounts for richness patterns [19]. Of the climatic factors, temperature and precipitation are commonly studied in a wide variety of animal and plant taxa research. Temperature may determine species richness of organisms through its effect on the biochemical kinetics of metabolism [20,21] or covarying with net primary productivity (NPP) [22,23]. Water–energy dynamics also play a key role in explaining globally extensive plant and animal diversity gradients, since water availability and optimal energy conditions are fundamental to biotic dynamics [17].

For the spatial patterns of species turnover or beta diversity, all explanations or hypotheses could be basically categorized into two broad families: niche-based assembly mechanism and neutral mechanism. The niche-based assembly mechanism focuses on environmental filtering processes, and thus patterns of species distributions are simply determined by environmental divergence [24,25]. In contrast, the neutral mechanism emphasizes the role of spatial processes across the landscape in shaping the composition of communities, and the geographical distance (or dispersal limitation) determines the species distributions [26]. Recently, Soberón (2010) and Hortal et al. (2010) further defined abiotic and biotic factors into four important elements, scenopoetic factors (e.g., temperature, precipitation, water-energy balance), bionomic factors (e.g., habitat heterogeneity), biogeographic factors and occupancy dynamics [27,28]. Based on their arguments, scenopoetic and biogeographic factors are fundamental at large scales, and bionomic effects and occupancy dynamics play more important roles at smaller scales [28].

Here, we aim to evaluate the influence and relative importance of spatial and local environmental factors in explaining variations in species richness and composition of epigaeic beetles along a geographic gradient in the Inner Mongolia grassland, North China. We selected spatial measures (longitude and latitude), scenopoetic measures (e.g., climate: temperature, precipitation, water-energy balance, harshness of conditions), and bionomic measure (environmental heterogeneity) to test the following questions:

(1) Which environmental variables determined richness and abundance patterns?

Firstly, we predict that climates play a more important role in determining richness and abundance than the other factors. Secondly, we predict that local variation in temperature, precipitation and water-energy balance might have a strong explanatory power in the local variation in richness and abundance of beetles.

(2) How did environmental variables affect species composition?

We partitioned the effects of spatial and local environmental components on the species distributions. Firstly, we test hypotheses about the processes (neutral and environmental filtering) that may be responsible for species distributions. Secondly, we predict that climatic factors play more important roles on community structure at the regional scale across 2000 km than do environmental heterogeneity.

## Material and Methods

### Study area and experimental design

**Fig 1:**
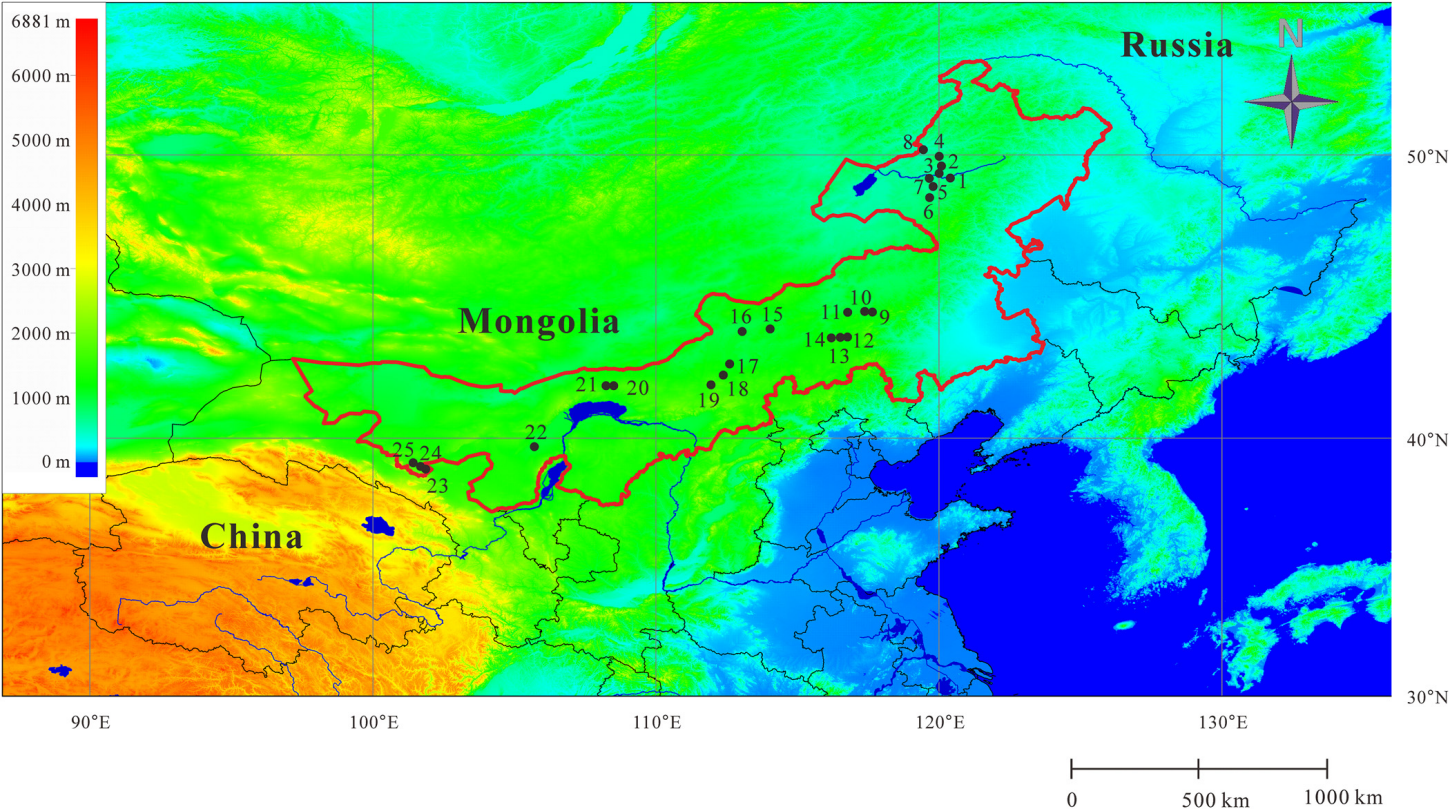
Map of the study area. The map shows the location of our 25 sampling sites (black dots) in the Inner Mongolia grassland (the area circled by red line) of North China.

This study was conducted in the Inner Mongolia grassland (87 million ha), the eastern part of the Eurasian steppe (Fig 1). The Inner Mongolia grassland belongs to the temperate steppe region, and is the largest contiguous biome in the world [29]. From east to west, the grasslands are dominated by meadow steppe, typical steppe, desert steppe and desert zones along a gradient of decreasing moisture [30].

Twenty-five natural arid and semiarid ecosystem sites were selected *a priori* to represent four typical steppe vegetation types (S1 Fig) on the Inner Mongolia Plateau along a 2000 km east–west transect (Fig 1). This transect runs from 38.91°N–50.19°N in latitude and 101.62°E–120.41°E in longitude, with elevation ranging from 530 m in the east to 1550 m in the west. Based on long-term meteorological data (1961–2000), the mean annual temperature ranges from -1.7°C to 8.6°C, and the mean annual precipitation is between 113.9 mm and 425.5 mm [31]. Eight sampling sites (Sites 1–8) were situated within the meadow steppe, 6 sites (Sites 9–14) within the typical steppe, 7 sites (Sites 15–21) within the desert steppe, and 4 sites (Site 22–25) within the desert (Fig 1). Detailed descriptions of climate, topography and flora can be found in Bai et al. (2008) [31].

The Inner Mongolia grassland has a long grazing history from both wild and domestic herbivores (primarily sheep, horses and cows in the recent century), and it is difficult to find a permanent undisturbed site. To preserve the grasses as the foods of the cattle in the winter, some temporary enclosures were usually fenced in the spring from large-animal grazing and were rarely disturbed over the growing season (hereafter referring to ‘lightly grazed’, see S2A Fig). In contrast, the outsides of the enclosures were frequently grazed by the cattle over the growing season (hereafter referring to ‘heavily grazed’, see S2B Fig). We took a strategy of reducing grazing pressure as much as possible to establish sampling plots in temporary enclosures. At each site, we established one or two sampling plots (500 m × 500 m). However, in some sites it was difficult to establish a lightly grazed sampling plot, so we also included some heavily-grazed sampling plots in this study. In total, we established 36 plots (14 lightly grazed plots and 22 heavily grazed plots) within the 25 sites in this study: 10 sites with 1 lightly grazed and 1 heavily grazed plot, 1 site with two lightly grazed plots, 2 sites with 1 lightly grazed plot alone, and the remaining 12 sites with one heavily grazed plot each only (S1 Table). For independence, the sampling plots were at least 500 m apart from each other [32,33,34].

### Beetle sampling

Beetles were sampled by pitfall traps. Although pitfall traps are biased toward actively moving species and inaccurate in estimating the absolute density, this method is useful in the monitoring and assessment of local population changes [35]. Within each plot, we set two transects, each of which was composed of 5 trapping locations. The distance between the two transects was 100 m or more, and trapping locations were 25 m apart from each other [36]. Each trapping location was composed of five traps. Within each trapping location, the five traps were 1m apart in a crossed pattern. Because of loss of some traps in 5 plots, a total of 1750 traps were used in this study (S1 Table). Traps were constructed from 400 mL plastic beverage cups (9 cm high by 7.5 diameter). Each trap was filled with about 100 mL of a mixed trapping fluid (vinegar:sugar:alcohol:water in the ratio of 10 ml:5 g:5 ml:20 ml) to collect beetles [37]. The trapped specimens were transferred to 70% alcohol. Beetle sampling was carried out during seasons of peak beetle foraging (mid-August to mid-September) in 2011 and 2012, corresponding to annual peak-standing biomass [31]. The traps were remained in operation for 48 h for each plot.

A total of 6025 epigaeic beetles were captured during this study with two families, Carabidae and Tenebrionidae, comprising more than 87% (5242 individuals) of the total catch. Thus, we only included these two families in our analysis. All specimens were deposited in the Insect Museum, Institute of Zoology, Chinese Academy of Sciences (CAS). Carabids were identified by Drs. Hong-Bin Liang and Hong-Liang Shi following the nomenclature by Lindroth (1961–1969) [38], and tenebrionids by Drs. Yi-Bin Ba and Feng-Yan Wang following the nomenclature by Ren & Yu (1999) [39]. A full species list was provided in S2 Table in Supporting Information.

### Environmental data

We obtained climate data from the WorldClim [40]. Based on a 30 arc-second resolution, we extracted temperature variables (mean annual temperature of the warmest quarter, temperature of the coldest quarter, temperature seasonality) and precipitation variables (mean annual precipitation, mean precipitation from April to October, precipitation seasonality) as well as data on frost frequency (number of days with temperature below 0°C), mean annual aridity (a numerical indicator of the degree of dryness of the climate at a given location), actual evapotranspiration (= AET) and potential evapotranspiration (= PET) (S3A–S3K Fig). For detailed calculation of AET and PET, see Yu et al. (2013) [13].

Through promoting the formation of small microhabitats at ground level and creating different environmental conditions, canopy cover, the related vegetation and soil properties can affect the abundance and distribution of epigaeic arthropods [41,42]. Moreover, canopy cover is easy to measure, compared with other variables associated with environmental heterogeneity (e.g., C:N:P stoichiometry, vegetation structure and soil properties, etc.). Therefore, we included the canopy cover (= canopy) as a surrogate of environmental heterogeneity into the analysis. The percentage data for the canopy cover was measured by visual estimation within a radius of 2 m around the center of each trapping location (S3L Fig).

### Data analysis

To reduce the possible bias from variable sampling sizes amongst the plots (S1 Table), we used sample-based rarefied richness to reduce the number of species to below the observed richness for plots with more samples [43]. Since incomplete sampling efforts also might result in a biased species number, we used the ratio between the observed number of beetle species and the expected number in each site to estimate the completeness of our beetle sampling [9]. The expected number was computed using the first-order jackknife richness estimator, a nonparametric estimator that performs relatively well under a wide range of sample sizes [44]. In this study, we rarefied back to 25 traps for each plot and computed jackknife richness estimate, using EstimateS 7.50 [45].

Some climatic variables that are highly correlated and multicollinearity might influence data analysis. Thus, we ran a collinearity diagnosis (Data reduction: Principal components analysis) to exclude variables with very low tolerance values due to high covariation with other model variables of the same set [46]. Among the dataset of climatic variables, precipitation from April to October, AET and annual aridity were highly correlated with precipitation, and temperature of the coldest quarter was strongly correlated with temperature, so we dropped these variables from the climate model (S3 Table).

We used ordinary least squares multiple linear regression models to analyze the potential of explanatory variables (space, climate, environmental heterogeneity) to predict patterns in richness and abundance. All variables included in the regression models were tested for normality prior to analysis. Data on abundance was square-root transformed to normalize model residuals, whereas the percentage data from canopy cover was arcsine transformed. Non-linear relationships between the response variables and explanatory variables were checked prior to model selection [46]. We calculated multiple-term regression models for different sets of predictors to assess independent as well as collective statistical effects of spatial, climatic and environmental heterogeneous factors [28,47]. Model selection was performed using Akaike’s information criterion (AIC) in the case of linear regression models and by stepwise backward elimination of non-significant variables from the models. We then used variation partitioning (partial regression analysis) to calculate independent and shared statistical effects of the models for space, climate and environmental heterogeneity on the geographic distribution of richness and abundance [28,48]. All statistical analyses were run in R 3.1.0 [49] and SAM 4.0 [50].

Geographical data were generally spatially autocorrelated, and thus can cause non-significant relationships to appear significant when using traditional statistical approaches. To correct for spatial autocorrelation in regression residuals, we assessed the potential effects of spatial autocorrelation in two ways following the method by Sanders et al. (2007) [21]. Firstly, we calculated Moran’s *I* across eight spatial distance classes for richness and abundance to test whether any of the response or predictor variables were spatially autocorrelated [51], using SAM 4.0 [50]. Secondly, to examine whether the residuals from the models for multiple regressions described above were spatially autocorrelated, we calculated Moran’s *I* on models that did not include spatial variables. If no spatial autocorrelation was found in the residuals of the model, then we conclude there was insignificant spatial autocorrelation [52].

Non-metric multidimensional scaling (NMDS) was used to describe and interpret the major gradients in beetle community data. In order to focus on compositional differences between plots independent of species-richness gradients and of variations in sampling effort among our plots, as Koleff et al. (2003) recommended [53], we applied Simpson index between samples to estimate the level of compositional similarity between pairs of sampling plots. NMDS was done using the software, PAST 3.0 [54].

As Borcard et al. (1992) suggested [55], we used canonical correspondence analysis (CCA) with Monte Carlo permutation tests of statistical significance to partition the variance in the beetle species composition dataset into pure spatial, pure environmental and spatially structured environmental fractions. To balance the effects of abundant and rare species on beetle composition, we performed two separate analyses: one for species presence or absence data and the other for abundance data. We used Monte Carlo permutations to evaluate the significance of each explanatory variable. Following the results of these tests, we excluded the variables of longitude, canopy cover, precipitation seasonality, temperature seasonality, temperature of the warmest quarter and annual temperature for presence or absence data, and canopy cover, temperature of the warmest quarter and precipitation seasonality for abundance data from our subsequent analyses. CCA was carried out using CANOCO 4.0 software [56].

We used simple linear regressions to analyze the relationship between geographic distance and similarity in species composition, known as the distance–decay in similarity relationship. Two datasets including presence/absence data and abundance data were considered. Simpson index was used to compute plot similarity for presence/absence data, whereas the Sørensen–Chao index was measured for plot similarity based on the matrix of abundance data [57]. Akaike’s information criterion was used to determine the best-fit regression model (linear–linear, log–linear, or log–log). We used simple Mantel tests to test the significance of each distance-similarity relationship (9999 permutations) with the software PASSAGE [58].

### Ethical considerations

All specimens used in this study were neither endangered nor protected species, and no specific permits were required for the described field studies.

## Results

We recorded 24 genera and 59 epigaeic beetle species from the 36 plots along the geographic gradient. A full list of these species is provided in S2 Table in Supporting Information. Of these species found, 36 species were identified to the family Carabidae, and 23 species to the family Tenebrionidae.

Most beetles occupied very narrow ranges along the longitudinal gradient (S4 Fig). Thirty-eight species (64.4%) were found at less than 5 plots, whereas only 8 species (13.6%) at more than 10 plots. In addition, there were 17 species (28.8%) found at only a single plot and 8 species (13.6%) at two plots throughout the study area. Three carabid species (*Cymindis binotata*, *Harpalus lumbaris*, *Poecilus gebleri*) occurred at more than 20 plots.

### Richness and abundance

**Fig 2:**
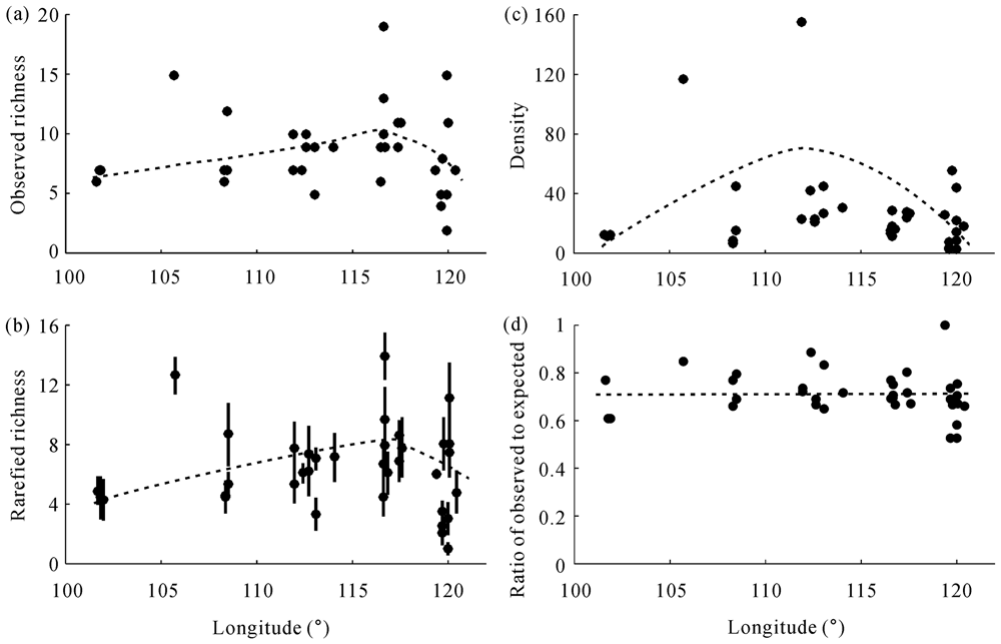
Geographic variation in beetle richness estimates and abundance along the longitudinal gradients in the Inner Mongolia grassland. (a) Observed richness, (b) Rarefied richness (rarefied to 25 traps) (with standard error), (c) abundance (density: mean number of individuals/25 traps), (d) ratio of observed to estimated (sampling completeness).

Rarefied richness was strongly correlated with observed richness (*r* = 0.975, n = 36, *P* < 0.001), showing similar hump-shaped patterns along the longitudinal gradient (Fig 2a and 2b). This reflected a steady increase in beetle richness from west to east, up to about 117°E, and from there a moderate decline in species richness (Fig 2a and 2b). Similarly, regression analyses using rarefied richness showed qualitatively similar results to those analyses using observed richness measures. We thus present results based on the rarefied richness values only. Beetle abundance also showed the similar hump-shaped pattern along the longitudinal gradient, but with the peak at 112°E (Fig 2c). The number of species recorded at each site represented 53%-100% (mean = 71.2%) of the number of species expected in those sites, and sampling completeness of captured species tended to be around the line of 0.70 value along the gradient (Fig 2d). This suggests that the possible bias from sampling incompleteness does not influence the pattern analysis of beetle richness and abundance along the longitudinal gradient.

The spatial regression models of richness and abundance also included latitude (Table 1), representing an increase in beetle richness and abundance from southern desert towards typical steppe (about 44°N) and then a decrease towards northern meadow steppe. The best spatial models (latitude and longitude) accounted for 17.2% and 24.0% of the variations in richness (AICc = 176.1 for the best model, compared to averaged AICc = 188.2 across 15 models) and abundance (AICc = 181.9 for best model, compared to averaged AICc = 198.1 across 15 models), respectively (Table 1).

**Table 1:** Regression models for geographic distribution of species richness and abundance.

Model type	Model (function)	R^2^	F	DF	*P*
Rarefied richness					
Space	-lat+long^2^	0.172	3.419	2, 33	0.045
Climate	PET^2^+prec-frost	0.309	4.760	3, 32	0.001
Complete	-lat+long^2^+PET^2^+prec-frost	0.333	2.992	5, 30	0.026
Abundance					
Space	long-long^2^	0.155	3.018	2, 33	0.063
Climate	-temp-temp^2^+warm-seast+PET^2^	0.352	3.254	5,30	0.018
Environmental heterogeneity	-canopy+canopy^2^	0.199	4.088	2, 33	0.026
Complete	long-long^2^-lat+lat^2^-temp-temp^2^+warm-seast+PET^2^-canopy+canopy^2^	0.597	3.233	11,24	0.008

The functions consist of single and/or polynomial terms (e.g., ‘factor–factor^2^’ or ‘factor+factor^2^’). ‘+’ indicates positive linear, ‘–’ negative linear relationships. Abbreviations of the variables: latitude (lat), longitude (long), mean annual temperature (temp), temperature of the warmest quarter (warm), temperature seasonality (seast), mean annual precipitation (prec), potential evapotranspiration (PET), frost frequency (frost), canopy cover (canopy).

Climate models had relatively high explanatory values in richness (R^2^ = 0.31) and abundance (R^2^ = 0.35) (Table 1). The best climate models to richness (AICc = 171.5 for the best model, compared to averaged AICc = 292.8 across 16383 models) included measure of precipitation (prec) as well as measures representing water-energy balance (PET) and harshness of climate (frost). More species was associated with high levels of precipitation and water–energy balance, and short frost periods. For abundance, temperature-related variables such as temperature (temp), temperature of the warmest quarter (warm), temperature seasonality (seast), and water-energy balance (PET) were included into the best model (AICc = 178.3 for the best model, compared to averaged AICc = 244.9 across 16383 models).

Environmental heterogeneity (canopy cover) did not show any effect on species richness, but had a significant relationship with abundance (R^2^ = 0.20; AICc = 179.8 for the best model, compared to averaged AICc = 181.2 across 3 models) (Table 1). More beetle individuals were captured in the plots covered with sparse or dense herbs, and the lower values occurred in the plots with the middle coverage of canopy cover (c.a. 40–50%).

**Fig 3:**
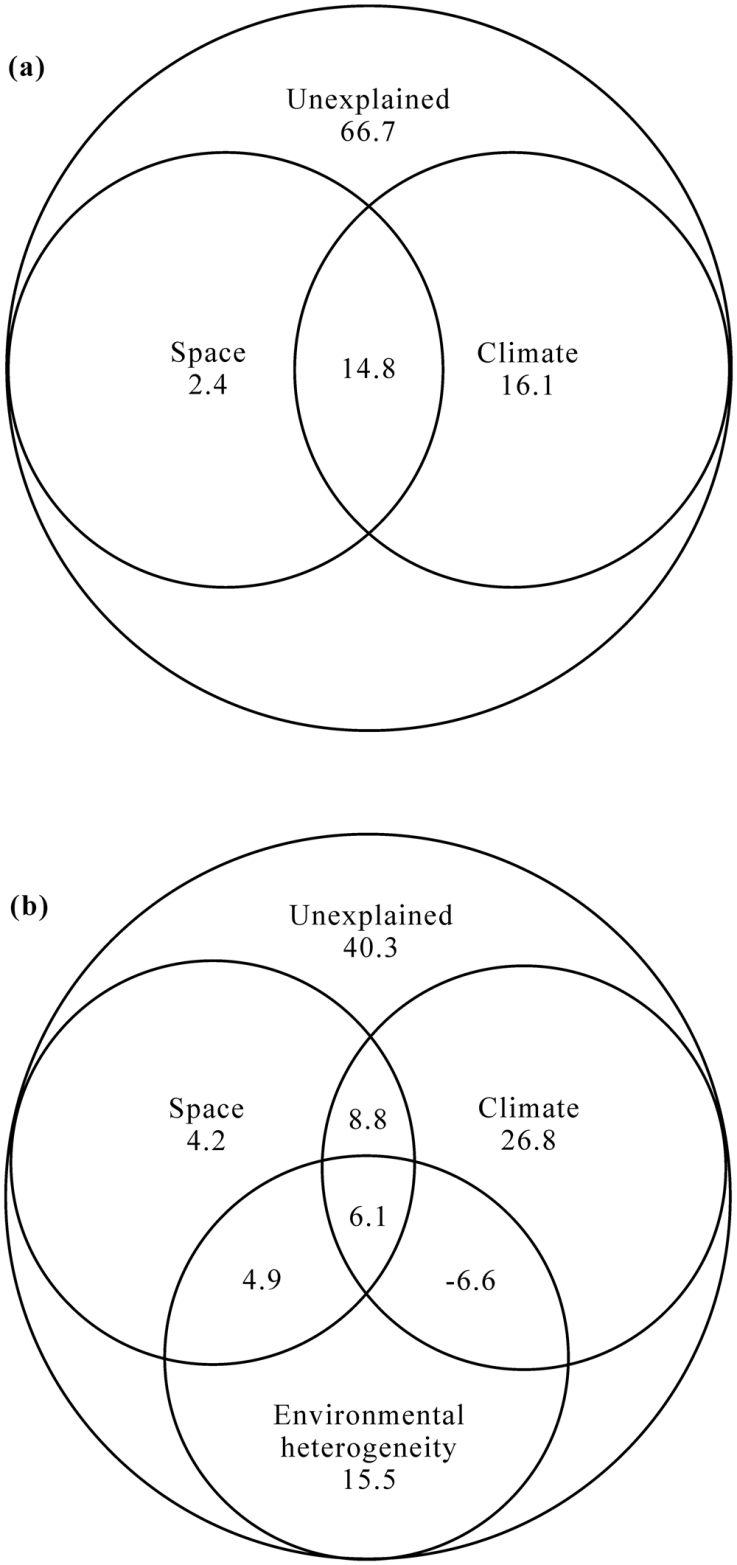
Variation partitioning for (a) species richness and (b) abundance (density) between independent and shared effects of regression models. Explanatory variables include space, climate and environmental heterogeneity.

Together, the examined factors (space, climate and environmental heterogeneity) explained 33.3% and 59.7% of data variability in richness and abundance, respectively. Variation partitioning indicated the strongest independent effects on richness and abundance to be climate (R^2^ = 0.161 and 0.268, respectively; Fig 3a and 3b). Environmental heterogeneity alone explained 15.5% of variation in abundance pattern (Fig 3b). In contrast, spatial variables alone explained negligible amount of variation in richness and abundance (only 2.4% and 4.2%, respectively; Fig 3a and 3b). In addition, 14.8% and 14.9% of variations in richness and abundance were observed in spatially structured climatic conditions (shared variation between space and climate), respectively (Fig 3a and 3b).

Little evidence of spatial autocorrelation was found in three estimates of richness and abundance (S4 Table). Fitting the models of richness and abundance patterns including spatial and local environmental variables removed all of the significant spatial autocorrelation in richness and abundance data across all distance classes (S5 Table).

### Species turnover

**Fig 4:**
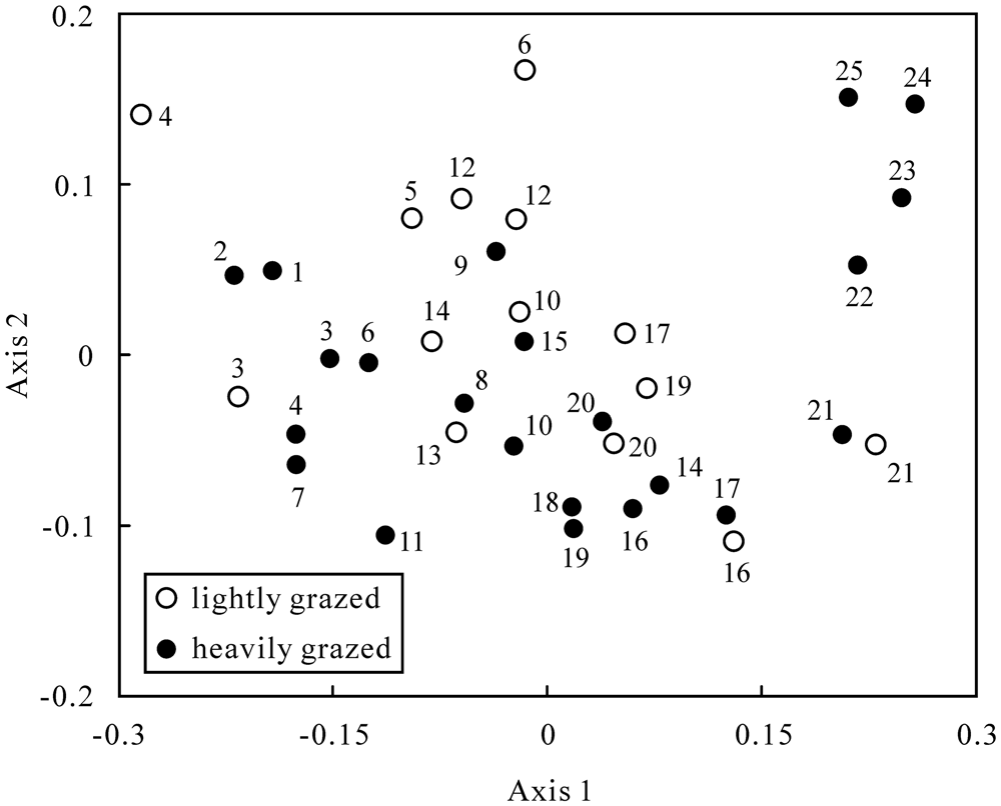
Non-metric multidimensional scaling (NMDS) ordination of the 25 sampling sites. Similarity in beetle species composition were based on Simpson index for presence or absence data. Site numbers are as in Fig 1.

The two-dimensional NMDS ordination explained 80.1% of total variance (stress = 0.23) and revealed a geographic/environmental gradient in species composition among survey sites (Fig 4). Axis 1 described a geographic gradient in precipitation, temperature and water–energy balance, with sites at the left side of the ordination plot being those with more rainfall, lower temperature and water–energy balance, whereas axis 2 might describe a precipitation seasonality gradient (Table 2). In particular, Axis 1 discriminated the five most southwestern sites (21–25) from the remaining sites: these sites were associated with a dry and hot desert environment (S3A, S3E and S3K Fig). Axis 2 further separated the four most southern sites (22–25) associated with lower precipitation seasonality from the others (S3G Fig). However, the heavily grazed sites cannot be discriminated from the lightly grazed sites in the ordination space (Fig 4).

**Table 2:** Pearson correlations.

	NMDS axis 1	NMDS axis 2
Longitude	-0.897 *	-0.089
Latitude	-0.862 *	0.034
Temperature	0.916 *	0.029
Potential evapotranspiration	0.889 *	0.122
Precipitation	-0.925 *	0.097
Precipitation seasonality	-0.525	-0.508 **

The correlations were analyzed between environmental and geographic variables and the non-metric multidimensional scaling (NMDS) ordination scores (for a two-dimensional ordination of the 36 sampling plots according to their similarity in epigaeic beetle species composition).

*P < 0.05

**P < 0.01.

**Fig 5:**
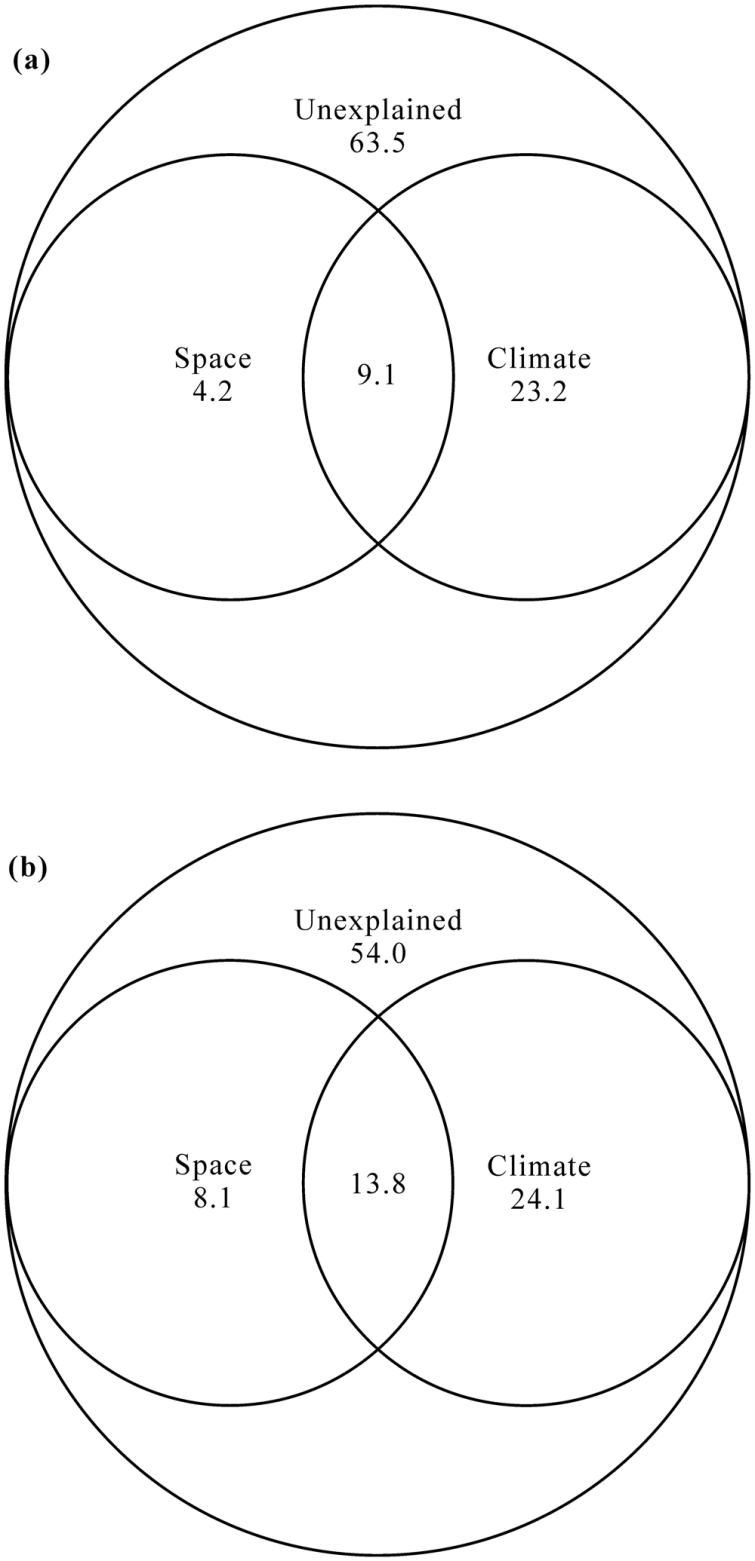
Variation partitioning for species composition using (a) presence/absence data and (b) abundance data between independent and shared effects of regression models. Explanatory variables include space, climate and environmental heterogeneity.

The CCA explained 36.5% of the total variance in the beetle community presence/absence data (pseudo-*F* = 3.454, *P* = 0.0002), suggesting a significant influence of climatic and spatial variables on beetle species composition (Fig 5a). Of the total variance in the beetle community data explained by the CCA, 63.6% was purely climatic (pseudo-*F* = 2.744, *P* = 0.0002), and 11.4% was purely spatial (pseudo-*F* = 1.963, *P* = 0.001); the interaction between these two sets of variables accounted for 25.0% of the explained variation. Comparable results were obtained when analyses were performed using abundance data with climatic factors still being the most important determinant of species composition (Fig 5b). Here, the total variance in the beetle community data explained by the CCA was 46.0% (pseudo-*F* = 3.403, *P* < 0.001) (Fig 5b), of which 52.4% was purely climatic (pseudo-*F* = 2.494, *P* < 0.001), 17.6% was purely spatial (pseudo-*F* = 2.093, *P* < 0.001) and 30.0% was the interaction of the two.

**Fig 6:**
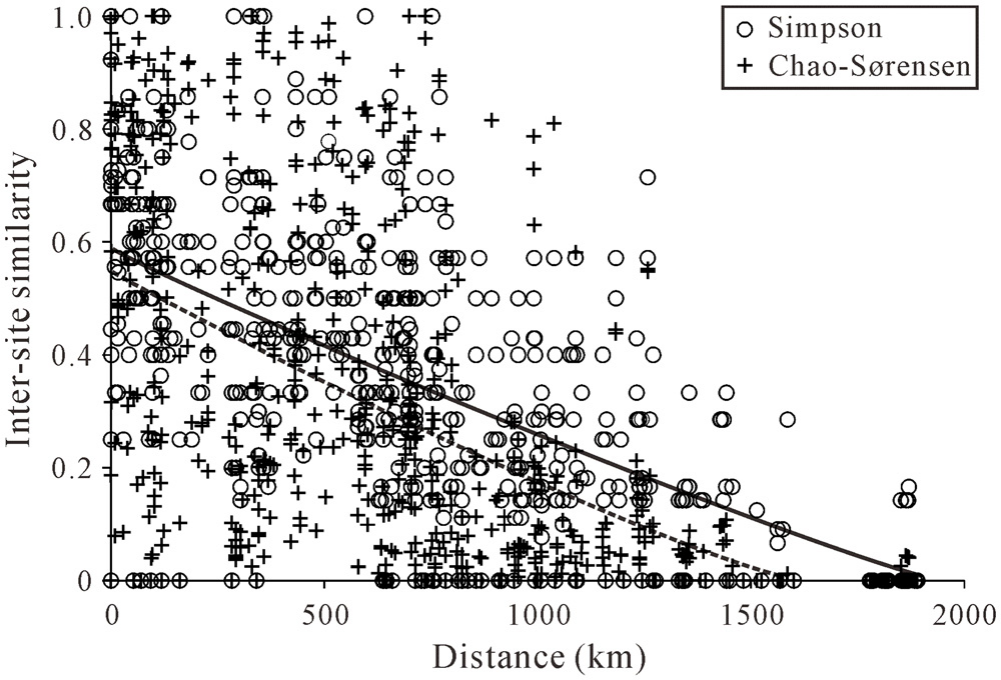
Similarity of beetle assemblages from the Inner Mongolia grassland as a function of distance between sampling sites. The lines represent the log-linear regression curves (continuous line = Simpson index of similarity for presence or absence data; dashed line = Chao–Sørensen index for abundance data).

When presence/absence data were considered, the average community similarity measured by the Simpson index was 0.36, whereas similarity by the Chao–Sørensen index with abundance data among plots was slightly lower (mean = 0.31), indicating that many plots did not share the same dominant species. Similarity between sites declined significantly as geographic distance increased (*r* = –0.629 and *r* = –0.575 for the Simpson and Chao–Sørensen indices, respectively; Mantel test, *P* < 0.001 in both cases; Fig 6). The relationship was best described by a log–linear regression for Simpson index (Log Simpson index = 0.199–0.0001·distance; adjusted *r*^*2*^ = 0.409) and for Chao–Sørensen index (Log Chao–Sørensen index = 0.189–0.0001·Distance; adjusted *r*^*2*^ = 0.356).

## Discussion

### Richness and abundance

Our study indicates that climate can explain a substantial amount of the variation in alpha diversity of epigaeic beetles over large geographic areas, as the two important reviews proposed [17,19]. Moreover, the interaction between climate with space or environmental heterogeneity also accounted for a high proportion of the explained variations. There is a longitudinal gradient in most climatic variables, especially for precipitation, temperature and PET, in the grassland (S3 Fig), and our results indicate that much of the observed variation in richness and abundance were explained by this gradient. These results on richness patterns also reinforced the idea that the interaction between water and energy, either directly or indirectly (via plant productivity), provides a strong explanation for globally extensive plant and animal diversity gradients [17], consistent with previous studies on epigaeic insects [9,23,59,60]. In contrast, temperature variables played important roles in accounting for the variation in abundance patterns, supporting the hypothesis of thermal limitations [22]. In addition, spatial autocorrelation analysis also confirms that spatial, climatic and environmental heterogeneous variables, especially for variables associated with temperature, precipitation and canopy cover, drive the geographic diversity gradients in epigaeic beetles.

Our finding also demonstrate that as a surrogate of environmental heterogeneity, canopy cover, had the strongest independent effect on beetle abundance patterns, consistent with previous studies [34,59,60]. In addition, to some extent, vegetation cover is also often correlated with above-ground net primary productivity (NPP) or total plant biomass [61,62], and thus it was used as a surrogate for plant productivity in some studies [59,60]. Although we did not obtain the direct measures of NPP or biomass, we could extract some NPP data from a previous study in the same study region [31]. This showed a significantly positive correlation between canopy cover in our study and NPP (r^2^ = 0.702, *P* < 0.001). We found no evidence, though, in support of the productivity hypothesis [22,63], when we analyzed the relationships between productivity and beetle diversity patterns (productivity model, richness: r^2^ < 0.01, *P* = 0.988; abundance: r^2^ = 0.03, *P* = 0.580) using the 14 extracted NPP data from Bai et al. (2008) [31] (S1 Table).

The influence of pure spatial factors was minimal on richness and abundance (only 2.4% and 4.2%) (Fig 3). However, 14.8% of the observed variation in richness was accounted for by the shared contribution of spatial and climatic factors, and 6.1% of the observed variation in abundance by the shared contribution of spatial, climatic and environmental heterogeneous factors. These shared contributions probably reflect the strong association between precipitation, PET, frost frequency, temperature, and longitude and latitude.

### Species composition

Of the potential explanatory factors we measured, most of them had statistically significant influence on the composition of local communities and accounted for a substantial amount of the variation. Our study illustrates the roles of spatial and local environmental components on the species distributions, and suggests that niche and neutral processes are not competing, and they actually worked together to shape beetle communities along a geographic gradient, consistent with previous findings [9,64]. However, similar to the ant study in Amazonian forests [9], spatial variables (longitude and latitude) accounted for less than 9% of the variations in beetle composition in this study, suggesting that neutral process contributed a comparatively smaller role to organizing beetle communities than do niche-based processes. The low rate of distance decay in community similarity also reinforced this point (Fig 6).

Our findings also support the Soberón’s hypothesis about species distributions across spatial scales [27], suggesting that climates played more important roles on community structure at the larger scale, other than biotic factor (environmental heterogeneity). According to the NMDS ordination (Fig 4), the sites were arranged from east to west along the longitude, corresponding to the gradients of precipitation, PET, precipitation seasonality and temperature (S3 Fig). Moreover, the four western desert sites (22–25) were clearly discriminated from the remaining sites, which might be attributed to the low values of the precipitation seasonality (S3G Fig). The ordination also reflected the characteristics of the studied beetles: tenebrionids usually abounded in sparse and drier sites (desert and desert steppe) [39], and carabids were mainly distributed in relatively dense and wetter sites (typical and meadow steppe) [65,66] (S4 Fig).

The heavily grazed sites cannot be discriminated from the lightly grazed sites in the ordination space (Fig 4), suggesting that environmental heterogeneity (canopy cover) resulting from grazing did not significantly affect the species composition at a larger scale. These findings were different from previous studies at a local or landscape scale [67,68], indicating that the influence of biotic factors becomes progressively more important as scale decreases [69].

In conclusion, our results suggest that spatial factors can work together with local environmental factors to shape epigaeic beetle communities along the geographic gradient in the Inner Mongolia grassland. Moreover, the climate features, especially precipitation, water-energy balance and temperature, and the interaction between climate with space and environmental heterogeneity appeared to play important roles in accounting for the shape of epigaeic beetles along the gradient.

## Supporting Information

S1 Fig Typical vegetation types in Inner Mongolia grassland. (PDF)

S2 Fig Lightly-grazed (A) and heavily-grazed (B) sampling plots in our study. (PDF)

S3 Fig Environmental variables considered for this study. (PDF)

S4 Fig Longitudinal range sizes of epigaeic beetles along the geographic gradients in the Inner Mongolia grassland. (PDF)

S1 Table Longitude, latitude, vegetation type, grazing gradient, aboveground net primary production (ANPP) and sampling efforts (trapping locations and traps) at 25 studied sites. (PDF)

S2 Table Composition and distribution of epigaeic beetles among four vegetation types along the geographic gradient in the Inner Mongolia grassland. (PDF)

S3 Table Reduction of dimension (Principal component analysis): factor analysis reduced variability within climatic variables to two dimensions. (PDF)

S4 Table Tests of spatial autocorrelation on the beetle diversity at species richness (observed and rarefied) and abundance. (PDF)

S5 Table Tests of spatial autocorrelation on the residuals of the multiple regression model on species richness (rarefied) and abundance. (PDF)
