# Supplementary material for: The Relative Importance of Spatial and Local Environmental Factors in Determining Beetle Assemblages in the Inner Mongolia Grassland
Source: PLoS One. 2016 May 3;11(5):e0154659. doi: 10.1371/journal.pone.0154659 (PMC4854484; doi:10.1371/journal.pone.0154659)
Supplement: S1 Fig — (PDF) [file pone.0154659.s001.pdf]

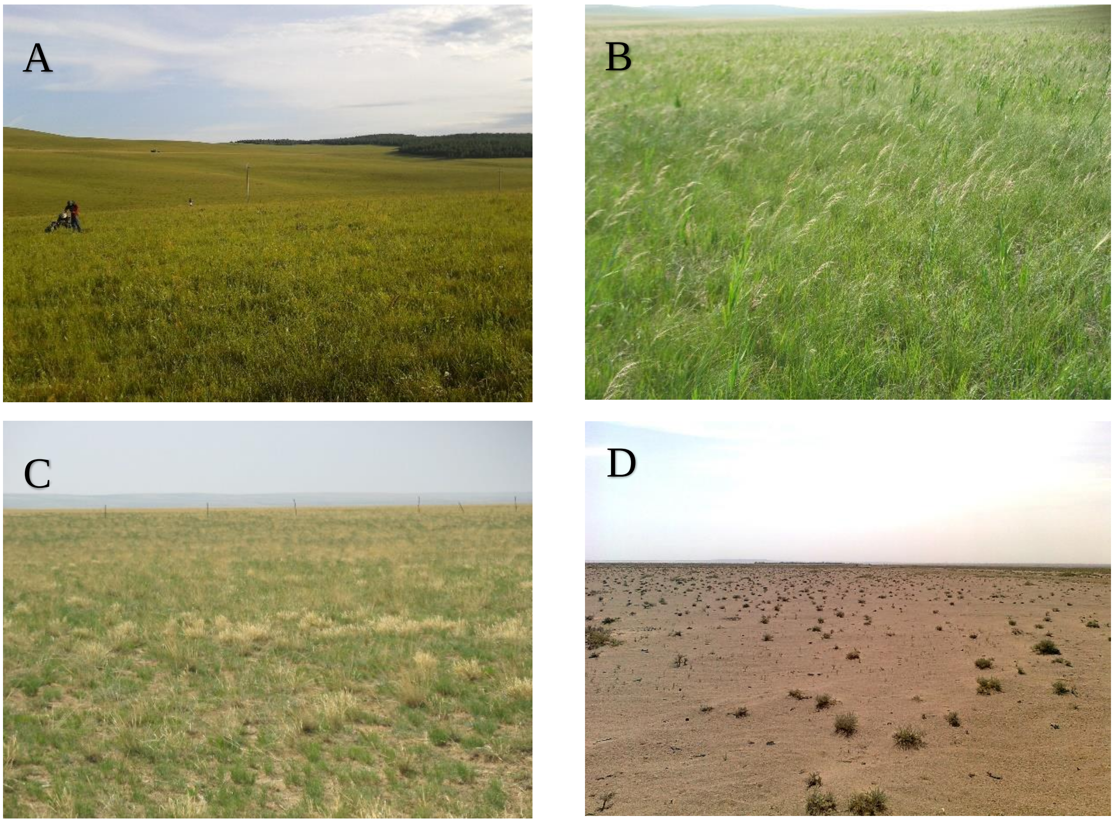

S1 Fig. Typical vegetation types in Inner Mongolia grassland. A: meadow steppe, B: typical steppe, C: desert steppe, D: desert
