# Supplementary figures and images for: The Relative Importance of Spatial and Local Environmental Factors in Determining Beetle Assemblages in the Inner Mongolia Grassland
Source: PLoS One. 2016 May 3;11(5):e0154659. doi: 10.1371/journal.pone.0154659 (PMC4854484; doi:10.1371/journal.pone.0154659)

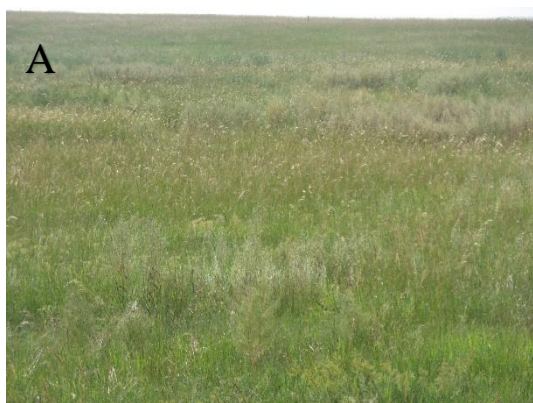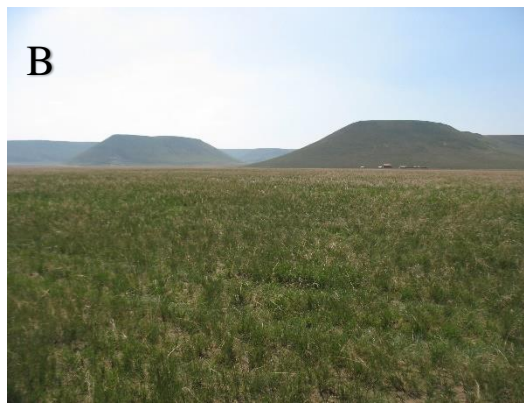

**S2 Fig. Lightly-grazed (A) and heavily-grazed (B) sampling plots in our study.**

Supplement: S2 Fig — (PDF) [file pone.0154659.s002.pdf]
