# Supplementary material for: The Relative Importance of Spatial and Local Environmental Factors in Determining Beetle Assemblages in the Inner Mongolia Grassland
Source: PLoS One. 2016 May 3;11(5):e0154659. doi: 10.1371/journal.pone.0154659 (PMC4854484; doi:10.1371/journal.pone.0154659)
Supplement: S3 Fig — (PDF) [file pone.0154659.s003.pdf]

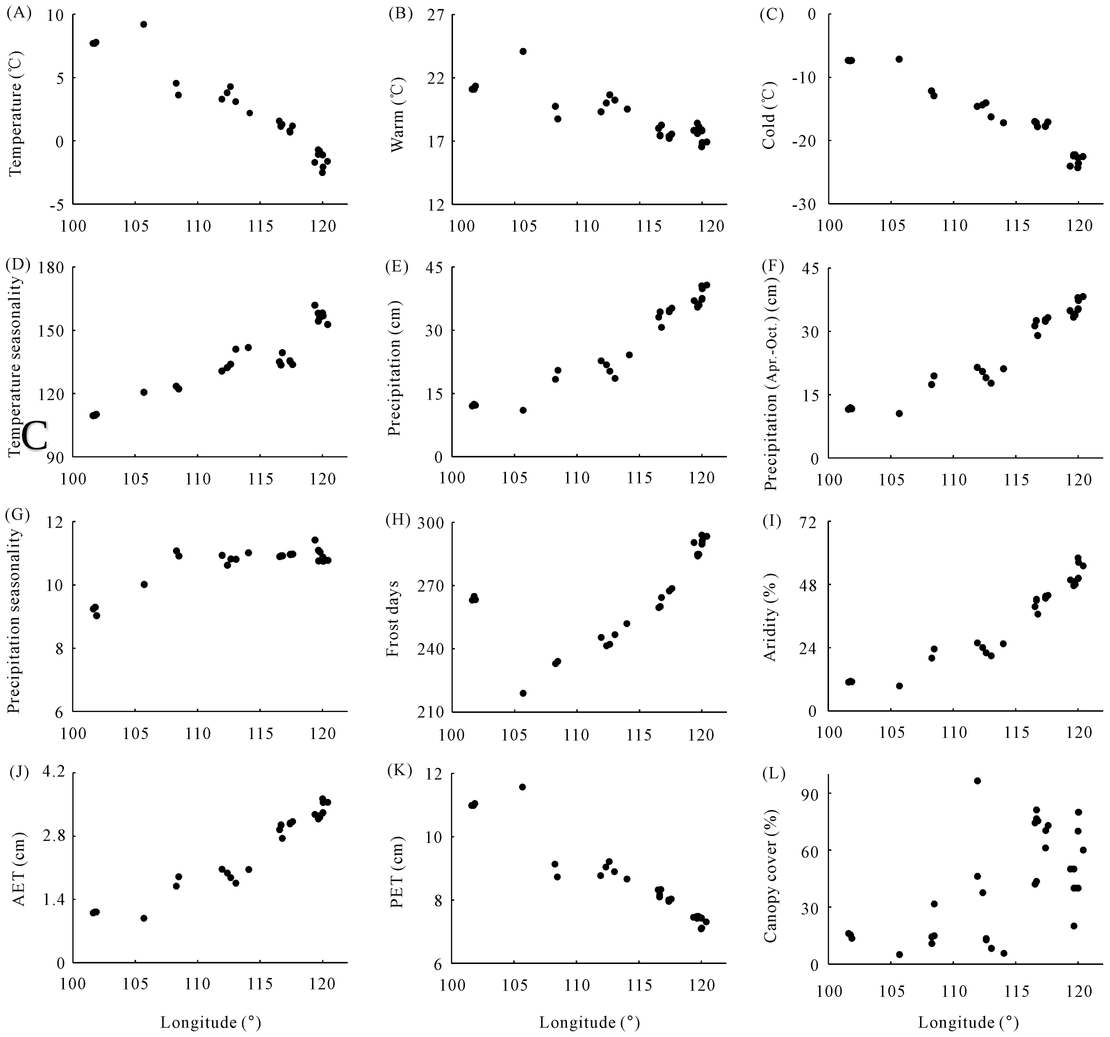

S3 Fig. Environmental variables considered for this study. (A) Mean annual temperature (temperature), (B) Temperature of the warmest quarter (warm), (C) Temperature of the coldest quarter (cold), (D) Temperature seasonality, (E) Mean annual precipitation (precipitation), (F) Mean precipitation from April to October (precipitation_Apr.-Oct.), (G) Precipitation seasonality, (H) Frost frequency (frost days), (I) Mean annual aridity (aridity), (J) Actual evapotranspiration (AET), (K) Potential evapotranspiration (PET), (L) Canopy cover.
