# Supplementary material for: The Relative Importance of Spatial and Local Environmental Factors in Determining Beetle Assemblages in the Inner Mongolia Grassland
Source: PLoS One. 2016 May 3;11(5):e0154659. doi: 10.1371/journal.pone.0154659 (PMC4854484; doi:10.1371/journal.pone.0154659)
Supplement: S1 Table — (PDF) [file pone.0154659.s005.pdf]

S1 Table. Longitude, latitude, vegetation type, grazing gradient, aboveground net primary production (ANPP) and sampling efforts (trapping locations and traps) at 25 studied sites.

Site#	Longitude	Latitude	Vegetation type	Grazing gradient	Trapping locations	Traps	ANPP*
1	E120°24.595'	N49°07.444'	Meadow steppe	heavily	10	50	
2	E120°02.921'	N49°31.625'	Meadow steppe	heavily	10	50	
3A	E120°01.658'	N49°22.480'	Meadow steppe	lightly	10	50	266.92
3B	E120°01.923'	N49°22.626'	Meadow steppe	heavily	10	50	
4A	E120°00.166'	N49°55.752'	Meadow steppe	lightly	10	50	261
4B	E120°00.155'	N49°55.716'	Meadow steppe	heavily	10	50	
5	E119°46.604'	N48°52.224'	Meadow steppe	lightly	5	25	229.61
6A	E119°40.201'	N48°29.641'	Meadow steppe	lightly	10	50	
6B	E119°40.169'	N48°29.598'	Meadow steppe	heavily	10	50	
7	E119°39.929'	N49°07.438'	Meadow steppe	heavily	10	50	
8	E119°23.089'	N50°11.189'	Meadow steppe	heavily	10	50	
9	E117°36.04'	N44°26.375'	Typical steppe	heavily	10	50	
10A	E117°24.740'	N44°27.477'	Typical steppe	lightly	9	45	194.3
10B	E117°23.731'	N44°27.731'	Typical steppe	heavily	10	50	
11	E116°46.731'	N44°25.478'	Typical steppe	heavily	10	50	192.51
12A	E116°40.576'	N43°33.091'	Typical steppe	lightly	10	50	
12B	E116°40.576'	N43°33.091'	Typical steppe	lightly	8	40	
13	E116°40.113'	N43°33.003'	Typical steppe	lightly	10	50	127.04
14A	E116°33.315'	N43°32.347'	Typical steppe	lightly	9	45	
14B	E116°33.262'	N43°32.272'	Typical steppe	heavily	9	45	
15	E114°03.17'	N43°50.842'	Typical steppe	heavily	10	50	
16A	E113°03.282'	N43°44.447'	Desert steppe	lightly	10	50	
16B	E113°03.201'	N43°44.429'	Desert steppe	heavily	10	50	
17A	E112°38.352'	N42°34.732'	Desert steppe	heavily	10	50	
17B	E112°38.352'	N42°34.732'	Desert steppe	lightly	10	50	
18	E112°22.345'	N42°12.282'	Desert steppe	heavily	10	50	
19A	E111°56.675'	N41°51.682'	Desert steppe	lightly	10	50	
19B	E111°56.675'	N41°51.682'	Desert steppe	heavily	10	50	
20A	E108°28.597'	N41°46.673'	Desert steppe	lightly	10	50	41.34
20B	E108°28.597'	N41°46.673'	Desert steppe	heavily	10	50	
21A	E108°17.422'	N41°49.757'	Desert steppe	lightly	10	50	59.68
21B	E108°17.422'	N41°49.757'	Desert steppe	heavily	10	50	
22	E105°41.817'	N39°41.242'	Desert	heavily	10	50	36.29
23	E105°41.817'	N39°41.242'	Desert	heavily	10	50	44.36
24	E101°46.312'	N38°56.103'	Desert	heavily	10	50	42.16
25	E101°37.427'	N39°01.38'	Desert	heavily	10	50	37.96

Site#: The number of sites corresponded to the location in Fig.1, and A and B represented two plots situated at the same site.

ANPP*: data was extracted from Bai et al. (2008)
