# Supplementary material for: The Relative Importance of Spatial and Local Environmental Factors in Determining Beetle Assemblages in the Inner Mongolia Grassland
Source: PLoS One. 2016 May 3;11(5):e0154659. doi: 10.1371/journal.pone.0154659 (PMC4854484; doi:10.1371/journal.pone.0154659)
Supplement: S2 Table — (PDF) [file pone.0154659.s006.pdf]

S2 Table. Composition and distribution of epigaeic beetles among four vegetation types along the geographic gradient in the Inner Mongolia grassland.

Species	Vegetation type				
	Meadow steppe	Typical steppe	Desert steppe	Desert	total
Carabidae					
Amara brevicollis (Chaudoir)	0	5	0	0	5
Amara dux Tschitscherine	0	12	0	0	12
Amara fodinae Mannerheim	4	200	0	0	204
Amara majuscula Chaudoir	36	0	0	0	36
Amara sp.	1	0	0	0	1
Bembidion sp.	83	0	0	0	83
Callisthenes anthrax (Semenoo)	0	2	0	0	2
Calosoma denticolle Gebler	3	10	1	0	14
Carabus glyptopterus Fischer	0	16	46	0	62
Carabus kruberi Fischer-Waldheim	136	40	0	0	176
Carabus latreillei Fischer	1	0	0	0	1
Corsyra fusula (Fischer von Waldheim)	5	33	573	0	611
Cylindera mongolica (Faldermann)	0	1	6	0	7
Cymindis binotata (Fischer von Waldheim)	152	287	45	0	484
Cymindis sp.1	0	0	0	78	78
Cymindis sp.2	1	0	0	0	1
Harpalus amplicollis Menetries	0	8	6	0	14
Harpalus brevicornis Germar	3	9	0	0	12
Harpalus calceatus (Duftschmid)	2	7	1	0	10
Harpalus froelichii Sturm	40	56	0	0	96
Harpalus lumbaris Mannerheim	20	51	123	1	195
Harpalus macronotus Tschitscherine	7	5	0	0	12
Harpalus modestus Dejean	9	0	0	0	9
Harpalus optabilis Dejean	0	37	0	0	37
Harpalus pallidipennis Morawitz	16	0	0	0	16
Harpalus sp.	10	0	0	0	10
Masoreus sp.	9	0	0	0	9
Microlestes sp.	18	0	0	0	18
Poecilus fortipes (Chandoir)	3	23	0	0	26
Poecilus gebleri (Dejean)	230	187	43	0	460
Poecilus mongoliensis (Jedlicka)	0	0	95	0	95
Pseudophoxenus rugipennis (Faldermann)	9	46	40	1	96
Pseudotaphoxenus hauseri (Fedlicka)	0	0	1	0	1
Pseudotaphoxenus originalis Schaufuβ	0	0	0	64	64
Pseudotaphoxenus sp.	0	0	0	1	1
Reflexisphodrus marginipennis (Pairmaine)	5	18	338	7	368
Tenebrionidae					
Anatolica amoenula Teitter	0	0	16	18	34

Anatolica externecostata Fairmaire	0	46	0	0	46
Anatolica mucronata Reitter	0	0	0	12	12
Anatolica nureti Schuster et Reymond	0	0	5	0	5
Anatolica omnoensis Skopin	0	0	0	19	19
Anatolica potanini Reitter	0	0	0	47	47
Anatolica sternalis Reitter	0	0	17	12	29
Blaps darvidis Deyrolle	0	23	0	0	23
Blaps femorailis dedusula Skopin	0	11	2	74	87
Blaps femoralis femoralis Fischer-Wald.	0	1	402	0	403
Blaps gressoria (Reitter)	0	0	1	0	1
Blaps reflexa Gebler	3	3	0	0	6
Blaps rugosa Gebler	0	4	10	0	14
Crypticus rufipes Gebler	107	0	0	0	107
Crypticus sp.	4	0	0	0	4
Cyphogenia chinensis (Faldermann)	0	0	0	9	9
Melanesthes mongolica Csiki	0	0	1	0	1
Melanesthes psammophila Kaszab	0	0	4	0	4
Microdera globata (Faldermann)	0	17	185	4	206
Microderakraatzi alashanica Skopin	0	5	5	731	741
Penthicus kiritshenkoi (Reichardt)	0	0	0	1	1
Scytosoma pygmaeum (Gebler)	11	92	1	0	104
Stenotrigon zichyi (Csiki)	0	0	0	13	13
