# Supplementary material for: The Relative Importance of Spatial and Local Environmental Factors in Determining Beetle Assemblages in the Inner Mongolia Grassland
Source: PLoS One. 2016 May 3;11(5):e0154659. doi: 10.1371/journal.pone.0154659 (PMC4854484; doi:10.1371/journal.pone.0154659)
Supplement: S3 Table — (PDF) [file pone.0154659.s007.pdf]

S3 Table. Reduction of dimension (Principal component analysis): factor analysis reduced variability within climatic variables to two dimensions. High factor loadings in the same dimension indicate possible collinearity within the variable groups.

	Dimension 1	Dimension 2
Temperature	-0.994	0.034
Temperature of the warmest quarter	-0.917	0.016
Temperature of the coldest quarter	-0.974	0.020
Temperature seasonality	0.895	-0.021
Precipitation	0.981	0.069
Precipitation from April to October	0.980	0.068
Precipitation seasonality	0.638	-0.762
AET	0.986	0.050
PET	-0.973	0.181
Aridity	0.986	0.068
Frost frequency	0.800	0.570
