# Supplementary material for: The Relative Importance of Spatial and Local Environmental Factors in Determining Beetle Assemblages in the Inner Mongolia Grassland
Source: PLoS One. 2016 May 3;11(5):e0154659. doi: 10.1371/journal.pone.0154659 (PMC4854484; doi:10.1371/journal.pone.0154659)
Supplement: S4 Table — (PDF) [file pone.0154659.s008.pdf]

S4 Table. Tests of spatial autocorrelation on the beetle diversity at species richness (observed and rarefied) and abundance. According to Bonferroni adjustment, significant values were set as the critical α to 0.006 to correct for OLS regressions.

		Distance class							
		1	2	3	4	5	6	7	8
Observed	Moran's *I*	0.077	0.048	-0.087	-0.263	-0.060	-0.003	0.181	-0.105
	Probability	0.501	0.595	0.345	0.022	0.503	0.984	0.068	0.174
Rarefied	Moran's *I*	0.081	0.042	-0.082	-0.195	-0.103	-0.014	0.181	-0.112
	Probability	0.491	0.577	0.321	0.084	0.226	0.878	0.084	0.154
Abundance	Moran's *I*	0.051	0.066	-0.183	0.137	0.046	-0.269	-0.159	0.016
	Probability	0.651	0.477	0.086	0.176	0.597	0.022	0.106	0.168
