# Supplementary material for: The Relative Importance of Spatial and Local Environmental Factors in Determining Beetle Assemblages in the Inner Mongolia Grassland
Source: PLoS One. 2016 May 3;11(5):e0154659. doi: 10.1371/journal.pone.0154659 (PMC4854484; doi:10.1371/journal.pone.0154659)
Supplement: S5 Table — (PDF) [file pone.0154659.s009.pdf]

**S5 Table. Tests of spatial autocorrelation on the residuals of the multiple regression model on species richness (rarefied) and abundance.** Explanatory variables included non-spatial variables only. According to Bonferroni adjustment, significant values were set as the critical α to 0.006 to correct for ordinary multiple regressions.

| | | Distance class | | | | | | | |
|---|---|---|---|---|---|---|---|---|---|
| Model | | 1 | 2 | 3 | 4 | 5 | 6 | 7 | 8 |
| Rarefied richness | | | | | | | | | |
| Climate | Moran's *I* | -0.110 | -0.097 | -0.003 | -0.041 | 0.022 | -0.016 | -0.057 | 0.073 |
| | Probability | 0.314 | 0.299 | 0.966 | 0.637 | 0.765 | 0.851 | 0.512 | 0.273 |
| Abundance | | | | | | | | | |
| Climate | Moran's *I* | -0.049 | -0.175 | 0.03 | -0.055 | 0.026 | -0.061 | 0.044 | 0.008 |
| | Probability | 0.667 | 0.077 | 0.687 | 0.534 | 0.719 | 0.464 | 0.601 | 0.872 |
| Habitat * | Moran's *I* | -0.013 | -0.037 | -0.110 | 0.143 | 0.043 | -0.149 | -0.154 | 0.134 |
| | Probability | 0.911 | 0.670 | 0.212 | 0.152 | 0.575 | 0.140 | 0.125 | 0.112 |

Habitat*: Environmental heterogeneity
